# Supplementary material for: Physical and Chemical Characterisation of Conventional and Nano/Emulsions: Influence of Vegetable Oils from Different Origins
Source: Foods. 2022 Feb 25;11(5):681. doi: 10.3390/foods11050681 (PMC8908978; doi:10.3390/foods11050681)
Supplement: Supplementary file 1 [file foods-11-00681-s001.zip › foods-1595069-supplementary.pdf]

Table S1. Fatty acid composition of oils (% of total fatty acids).

Fatty Acid	Oil Type				
	EVOO	CPRO	OO	RO	SO
C8:0	ND	ND	ND	ND	ND
C10:0	ND	ND	ND	ND	ND
C12:0	ND	ND	ND	ND	ND
C14:0	ND	ND	ND	ND	ND
C16:0	1.10 (0.16)	ND	0.88 (0.01)	0.20 (0.01)	ND
C18:0	13.16 (0.47)	4.88 (0.03)	12.40 (0.02)	4.79 (0.02)	6.52 (0.13)
C18:1	77.69 (1.43)	74.39 (0.10)	79.73 (0.08)	74.11 (0.10)	31.95 (1.75)
C18:2	8.04 (0.86)	20.73 (0.11)	6.98 (0.08)	20.57 (0.10)	61.53 (1.63)
C18:3	ND	ND	ND	0.33 (0.01)	ND
SFA%	14.26 (0.59)	4.88 (0.03)	13.28 (0.02)	4.79 (0.02)	6.52 (0.13)
MUFA%	77.69 (1.43)	73.39 (0.10)	79.73 (0.08)	74.11 (0.10)	31.95 (1.75)
PUFA%	8.04 (0.86)	20.73 (0.11)	6.98 (0.08)	20.90 (0.09)	61.53 (1.63)
Total	100.00	100.00	100.00	100.00	100.00

Indicated values are reported as means (standard deviation). ND—not detectable.

Table S2. Colour of vegetable oils: extra virgin olive oil (EVOO), cold-pressed rapeseed oil (CPRO), olive oil (OO), rapeseed oil (RO), sunflower oil (SO).

Oils	L^*	a^*	b^*
EVOO	55.46 ^{c} (2.74)	−9.52 ^{e} (0.20)	69.00 ^{b} (4.07)
CPRO	58.90 ^{b} (0.55)	−7.46 ^{c} (0.03)	76.61 ^{a} (2.52)
OO	62.54 ^{a} (0.25)	−8.76 ^{d} (0.20)	43.46 ^{c} (2.00)
RO	64.06 ^{a} (1.95)	−3.65 ^{b} (0.10)	10.47 ^{d} (0.30)
SO	63.59 ^{a} (1.47)	−2.33 ^{a} (0.02)	6.67 ^{d} (0.04)

Indicated values are reported as means (standard deviation). Values with the different superscript letters (within same column) are significantly different ($p < 0.05$).

Table S3. Total phenolic content (TPC), free fatty acids (FFA), radical scavenging activity and thiobarbituric acid reactive substances (TBARS) of vegetable oils. Extra virgin olive oil (EVOO), cold-pressed rapeseed oil (CPRO), olive oil (OO), rapeseed oil (RO), sunflower oil (SO).

Oils	TPC (mg GAE/ kg Oil)	FFA (% Oleic Acid)	Radical Scavenging Activity (%)	TBARS (mmol/kg Oil)	Viscosity (mPa·s)	Density (g/mL)
EVOO	202.64 ^{a} (13.96)	0.312 ^{a} (0.073)	34.15^{a} (2.64)	0.158 ^{c} (0.007)	63.82 ^{d} (2.10)	0.9112 ^{d} (0.0002)
CPRO	50.43 ^{c} (11.71)	0.241 ^{b} (0.025)	3.97 ^{b} (0.23)	0.278 ^{a} (0.020)	58.45 ^{b} (2.90)	0.9143 ^{b} (0.0004)
OO	91.98 ^{b} (5.88)	0.221 ^{b} (0.012)	8.31 ^{b} (2.75)	0.200 ^{b} (0.013)	65.84 ^{cd} (1.60)	0.9122 ^{cd} (0.0016)
RO	85.14 ^{b} (11.71)	0.159 ^{c} (0.036)	31.79 ^{a} (3.46)	0.092 ^{d} (0.018)	57.72 ^{bc} (2.23)	0.9140 ^{bc} (0.0004)
SO	31.90 ^{d} (1.75)	0.153 ^{c} (0.023)	4.91 ^{b} (1.00)	0.011 ^{e} (0.002)	53.97 ^{a} (3.67)	0.9187 ^{a} (0.0013)

Indicated values are reported as means (standard deviation). Values with the different superscript letters (within same column) are significantly different ($p < 0.05$).
